# Brachial Tunneled Peripherally Inserted Central Catheters and the Risk of Catheter Complications: A Systematic Review and Meta-Analysis

**DOI:** 10.3390/nursrep14010035

**Published:** 2024-02-18

**Authors:** Davide Giustivi, Mattia Donadoni, Stefano Maria Elli, Francesco Casella, Massimiliano Quici, Chiara Cogliati, Silvia Cavalli, Giulia Rizzi, Leyla La Cava, Arianna Bartoli, Elena Martini, Alba Taino, Martina Perego, Antonella Foschi, Roberto Castelli, Maria Calloni, Antonio Gidaro

**Affiliations:** 1 Post-Anesthesia Care Unit ASST Lodi, 26900 Lodi, Italy; davide.giustivi@asst-lodi.it; 2 Department of Biomedical and Clinical Sciences “Luigi Sacco”, Luigi Sacco Hospital, University of Milan, 20122 Milan, Italy; donadoni.mattia@asst-fbf-sacco.it (M.D.); francesco.casella@asst-fbf-sacco.it (F.C.); quici.massimiliano@asst-fbf-sacco.it (M.Q.); chiara.cogliati@unimi.it (C.C.); giulia.rizzi@unimi.it (G.R.); leyla.lacava@unimi.it (L.L.C.); arianna.bartoli@unimi.it (A.B.); elena.martini@unimi.it (E.M.); alba.taino@asst-fbf-sacco.it (A.T.); martina.perego@unimi.it (M.P.); maria.calloni@asst-fbf-sacco.it (M.C.); 3 Healthcare Profession Department—PICC Team, University of Milan Bicocca, IRCCS San Gerardo dei Tintori Foundation Hospital, 20126 Monza, Italy; stefanomaria.elli@irccs-sangerardo.it (S.M.E.); silvia.cavalli@irccs-sangerardo.it (S.C.); 4 Department of Infectious Diseases, Luigi Sacco Hospital, 20157 Milan, Italy; foschi.antonella@asst-fbf-sacco.it; 5 Department of Medicine, Surgery and Pharmacy, University of Sassari, Viale San Pietro N° 8, 07100 Sassari, Italy; rcastelli@uniss.it

**Keywords:** Peripherally Inserted Central Catheter (PICC), tunnel, infection, catheter-related thrombosis (CRT), bleeding, wound oozing, dislodgment, medical adhesive-related skin injury (MARSI) nerve injuries, artery injuries

## Abstract

Introduction: Situations involving increased workloads and stress (i.e., the COVID-19 pandemic) underline the need for healthcare professionals to minimize patient complications. In the field of vascular access, tunneling techniques are a possible solution. This systematic review and meta-analysis aimed to compare the effectiveness of tunneled Peripherally Inserted Central Catheters (tPICCs) to conventional Peripherally Inserted Central Catheters (cPICCs) in terms of bleeding, overall success, procedural time, and late complications. Methods: Randomized controlled trials without language restrictions were searched using PUBMED^®^, EMBASE^®^, EBSCO^®^, CINAHL^®^, and the Cochrane Controlled Clinical Trials Register from August 2022 to August 2023. Five relevant papers (1238 patients) were included. Results: There were no significant differences in overall success and nerve or artery injuries between the two groups (*p* = 0.62 and *p* = 0.62, respectively), although cPICCs caused slightly less bleeding (0.23 mL) and had shorter procedural times (2.95 min). On the other hand, tPICCs had a significantly reduced risk of overall complications (*p* < 0.001; RR0.41 [0.31–0.54] CI 95%), catheter-related thrombosis (*p* < 0.001; RR0.35 [0.20–0.59] IC 95%), infection-triggering catheter removal (*p* < 0.001; RR0.33 [0.18–0.61] IC 95%), wound oozing (*p* < 0.001; RR0.49 [0.37–0.64] IC 95%), and dislodgement (*p* < 0.001; RR0.4 [0.31–0.54] CI 95%). Conclusions: The tunneling technique for brachial access appears to be safe concerning intra-procedural bleeding, overall success, and procedural time, and it is effective in reducing the risk of late complications associated with catheterization.

## 1. Introduction

The COVID-19 pandemic has highlighted how stressful conditions among healthcare workers can significantly increase complications related to venous access devices, particularly catheter-related infections. Therefore, all strategies for preventing complications are mandatory, even during the anticipation of possible similar events [1].

A survey carried out during the first wave in Lombardy found that patients with COVID-19 were at higher risk of catheter-related thrombosis (*p* < 0.001; OR = 2.00 [1.85–5.03] CI 95%), catheter-related bloodstream infections (*p* < 0.001; OR = 3.82 [1.82–8.97] CI 95%), and dislodgement (*p* < 0.001; OR = 2.39 [1.80–3.20] CI 95%) compared to patients without COVID-19 [2].

To reduce these complications, the tunneling technique may be a viable option to consider. The Center for Disease Control and Prevention’s 2011 guidelines for the prevention of intravascular catheter-related infections emphasizes that tunneled catheters have a lower rate of infections than non-tunneled ones [3]. The creation of a subcutaneous route to move the exit site from the puncture position to another point on the skin has grown from the early 1970s (after the papers by Broviac [4] and Hickman [5]) to the present day [6].

Currently, the tunneling technique is the standard procedure for the insertion of long-term Central Vascular Access Devices for dialysis, parenteral nutrition, and chemotherapy [7]. The first tunneled Peripherally Inserted Central Catheter (PICC) was reported in 2001 by Selby et al. [8]. At the same time, some limitations became apparent in the use of PICCs, such as the impossibility of inserting the catheter in patients with an unfavorable catheter/vein ratio (i.e., pediatric patients, large-bore catheters) or the need to place the exit site in unusual positions (i.e., in burned patients) [9]. More recently, some papers have addressed this issue in relation to both venipuncture and tunneling techniques [10,11] and have provided proactive planning techniques and approaches to vascular catheterization [12,13,14].

This systematic review and meta-analysis aims to investigate the safety profile (overall success, nerve or artery injury, bleeding during insertion, and procedural time) and the efficacy profile [infection-triggering catheter removal; both catheter-related bloodstream infection (CRBSI) and infection of the exit site/tunnel; catheter-related thrombosis (CRT) and dislodgement] when comparing tunneled PICCs (tPICCs) to conventional PICCs (cPICCs). The secondary aim was the evaluation of the risk of medical adhesive-related skin injury (MARSI) and wound oozing.

## 2. Materials and Methods

Our systematic review and meta-analysis was conducted following the Preferred Reporting Items for Systematic Reviews and Meta-analysis (PRISMA) guidelines [15] and was registered on PROSPERO (CRD42022370474).

### 2.1. PICOS Questions

We sought information from patients who had PICC insertions (P) about the effect of tPICCs (I) compared to cPICCs (C) in terms of safety during the procedure, including overall success, nerve or artery injuries, bleeding, and procedural time, and late complications, including infection-triggering catheter removal (both CRBSI and infection of the exit site/tunnel), CRT, dislodgment, wound oozing, and MARSI (O), enrolled in randomized controlled trials (S). 

### 2.2. Data Sources and Searches

From August 2022 to August 2023, randomized controlled trials (RCTs) without language restrictions were searched using PUBMED^®^, EMBASE^®^, EBSCO^®^, CINAHL^®^, and the Cochrane Controlled Clinical Trials Register. No time restriction was used during the research.

Search strings were developed with the assistance of a medical librarian and the MesH term browser [https://meshb.nlm.nih.gov/ (accessed on 31 March 2023)]. They contained terms and synonyms for “Peripherally Inserted Central Catheter” or “PICC”, and “tunnel” or “subcutaneous tunneling”. 

The research strategy is reported in the Supplementary Materials.

### 2.3. Study Selection

After removing duplicates, title/abstract and full-text screening was performed independently by two authors (A.G., M.D.) using pre-defined inclusion and exclusion criteria. All original RCTs comparing the efficacy of tPICCs to any other cPICCs for the risk of infection, CRT, bleeding, MARSI, and dislodgment were included. The exclusion criteria were as follows: (1) studies describing fewer than ten patients; (2) studies focusing on the insertion technique of tPICCs, instead of complications. Hand searches and snowballing finalized the search. When multiple publications of the same research group/center described potentially overlapping cohorts, the authors selected the most recent publications. Disagreements were resolved by discussion.

### 2.4. Data Extraction and Quality Assessment

Data extraction was independently performed by two authors (A.G., M.D.) who screened and selected the included studies through Covidence software (https://www.covidence.org/blog/release-notes-april-2022/, accessed on 24 September 2023). Any disagreement was resolved by discussion or involving a third review author (F.C.). The extracted data included the following: the number of cPICCs and tPICCs, the safety profile [overall success, nerve or artery injuries, bleeding during insertion (milliliters), and procedural time (minutes)] and the efficacy profile (infection triggering catheter removal, CRT, MARSI, wound oozing, and dislodgement). 

The methodological quality of the selected articles was assessed by an index that classified studies as adequate, inadequate, or unclear. The risk of bias per study was evaluated and double-checked by two authors (A.G., M.D.) using the Risk of Bias (RoB) 2.0 tool for RCTs [16,17]. Additionally, the applicability of the included studies was assessed per the PICOS (Population, Intervention, Comparator, Outcomes, and Setting) domains. Each domain was assessed for low (+), high (×), or moderate (?) applicability concerns [18].

### 2.5. Data Synthesis and Analysis

We summarized data statistically whenever possible. The statistical analysis was conducted following the statistical guidelines outlined in the most recent edition of the Cochrane Handbook for Systematic Reviews of Interventions [19]. We used Review Manager 5 for review production and data analysis. We used a fixed-elect model to pool data when statistical heterogeneity was not statistically significant; in instances where heterogeneity was significant, we used a random model.

We used a risk ratio (RR) with a 95% confidence interval (CI) and the number needed to treat for an additional beneficial outcome (NNTB) if a statistically significant result was obtained to measure any effect on dichotomous variables (i.e., overall success, nerve or artery injuries, infection triggering catheter removal, CRT, MARSI, wound oozing, and dislodgement). We calculated NNTB values from the RR according to the formula NNTB (or number needed to treat for an additional harmful outcome (NNTH)) = 1/ACR × (1 − RR), for which ACR is the assumed control risk [20]. 

For continuous variables, we employed the mean and standard deviation when available. If not available, we estimated them using the median and interquartile range according to the method outlined by Wan et al. [21].

### 2.6. Assessment of Heterogeneity 

We attempted to explain any relevant clinical, methodological, or statistical heterogeneity using the I^2^ statistic [20]. Heterogeneity across the studies was assessed through both Q and I^2^ tests, which were considered significant when the *p*-value was <0.05 and I^2^ > 75% [22]. 

## 3. Results

**Figure 1:**
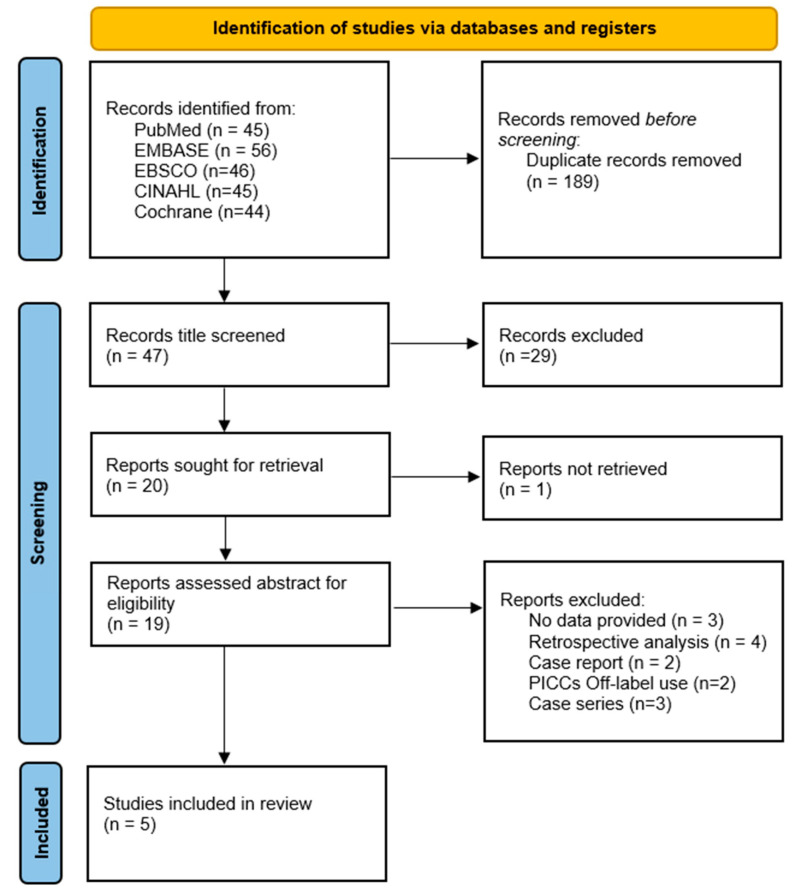
PRISMA 2020 flow diagram.

The search identified 236 articles (Figure 1). Following the removal of duplicates, 47 articles were initially identified during the title-screening process. However, only 19 of these were further assessed by analyzing their abstracts. One article was excluded following a thorough examination of the full text because it consisted solely of a protocol of RCT without any actual outcomes [23]. Five reports were excluded because they were more focused on the technique description rather than presenting results [8,9,10,12,13]. Two studies were excluded because of their retrospective design [24,25]. Four studies were ruled out because they described an off-label use of PICCs and did not refer to brachial access devices [26,27,28,29]. Other excluded studies included a case report of tunneled midline insertion [30] and a case series [11].

Eventually, five articles (1238 patients) were included in the qualitative and quantitative analysis. All the authors conducted an independent search on Medline checking for further evidence, and two explored all the references of the 5 studies [A.G., M.D.] (Table 1).

### 3.1. Study Characteristics

All studies were published in the last five years and were written in English. Four studies were conducted in Asia (China) [31,32,33,34] and one in Europe (Greece) [35]. Recruiting time ranged from 5 to 11 months. All the patients enrolled were outpatients with cancer disease (Table 1).

### 3.2. Risk of Bias and Applicability

**Figure 2:**
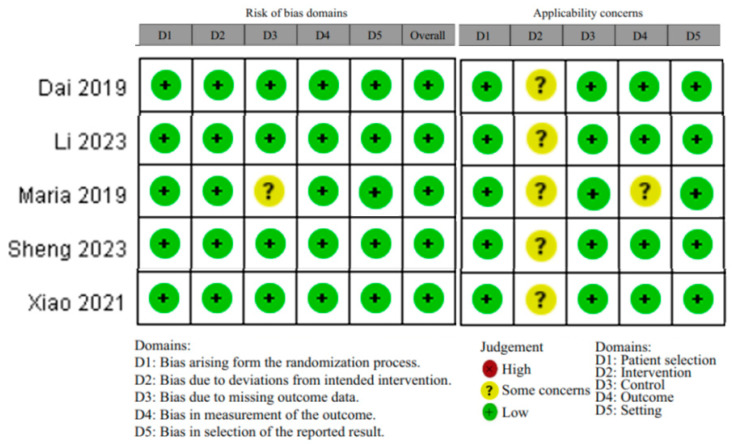
Risk of bias table, using the Risk of Bias (RoB) 2.0 tool for RCTs [31,32,33,34,35].

A ‘low’ risk of bias was observed in most of the studies (Figure 2). 

The applicability per patient selection, control, and setting domain was scored as ‘low’. 

The applicability per intervention domain was scored as ‘moderate’ due to a lack of uniformity in the control groups through the four studies (different materials of catheters, different techniques of creating the subcutaneous tunnel, and different ways to close the nick of the puncture site).

Since not all the outcomes were recorded, the applicability per outcome domain was scored as a ‘moderate’ concern for Maria et al. [35]. 

### 3.3. Results of Individual Studies

The first RCT published on this topic was the study conducted by Dai et al. [31] in 2019. The tPICC group showed a lower incidence of complications during the placement (18.4% vs. 32.2%; *p* = 0.036), a lower incidence of wound oozing (27.6% vs. 57.5%; *p* < 0.001), a lower incidence of medical adhesive-related skin injury (9.2% vs. 25.3%; *p* = 0.005), a lower incidence of venous thrombosis (1.1% vs. 9.2%; *p* = 0.034), and a lower incidence of catheter dislodgement (1.1% vs. 9.2%; *p* = 0.034).

Contrary to this, in the same year, Maria et al. [35] observed no significant differences in terms of complications when comparing cPICCs to tPICCs.

More recently, the study conducted by Xiao et al. [32] showed that tPICCs had a significantly lower occurrence of complications after placement, especially catheter dislodgement (3.1% vs. 15.4%; *p* = 0.03), CRT (3.1% vs. 15.4%; *p* = 0.03), and wound oozing (14.1% vs. 27.7%; *p* = 0.032). Also, tPICCs had a significantly lower occurrence of unscheduled PICC removal (3.1% vs. 13.8%; *p* = 0.029).

In the last year, Li et al. [33] reported that tunneled PICCs with tunnel lengths longer than 4 cm were associated with increased catheter dwell time and fewer PICC-related complications, including wound oozing, catheter dislodgement, and unplanned catheter removal.

Lastly, Sheng et al. [34] demonstrated that tPICCs significantly reduced the incidence of overall complications, particularly in terms of infection (3.0% vs. 7.1%; *p* = 0.021) and catheter-related thrombosis (3.3% vs. 8.3%; *p* = 0.008).

### 3.4. Results of Syntheses: Safety Profile

**Figure 3:**
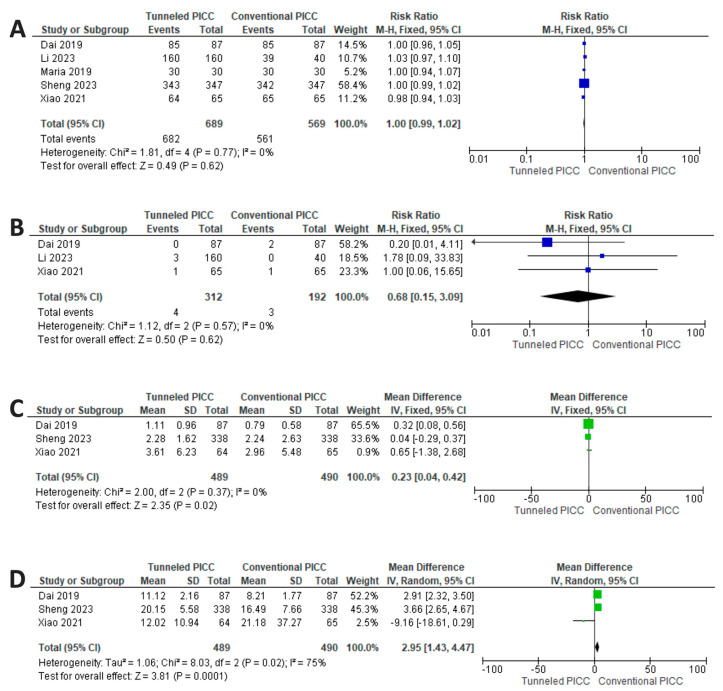
Results of synthesis. (**A**) No differences between cPICCs and tPICCs in overall success. (**B**) No differences between cPICCs and tPICCs in nerve or artery injuries. (**C**) Reduced risk for cPICCs for bleeding. (**D**) Reduced procedural time for cPICCs [31,32,33,34,35].

No difference can be observed in the overall success (defined as successful completion of procedure) when comparing tPICCS to cPICCS (*p* = 0.62; RR 1 [0.99–1.02] CI 95%) with a fixed model due to non-significant between-study heterogeneity (*p* = 0.77; I^2^ = 0%) (Figure 3).

No difference can be observed regarding nerve or artery injuries when comparing tPICCS to cPICCS (*p* = 0.50; RR 0.68 [0.15–3.06] CI 95%) with a fixed model due to non-significant between-study heterogeneity (*p* = 0.57; I^2^ = 0%) (Figure 3).

None of the studies examined reported major bleeding and hematoma formation. The amount of blood lost during the procedure (expressed in milliliters) is significantly greater for tPICCS (*p* = 0.02; RR = 0.23 [0.004–0.42] CI 95%), with a fixed model used due to the non-significant heterogeneity of the studies (*p* = 0.37; I^2^ = 0%) (Figure 3). tPICCs have a higher procedural time compared to cPICCs (*p* ≤ 0.001; RR 2.95 [1.43–4.47] CI 95%) (Figure 3). A random model was applied due to significant between-study heterogeneity (*p* = 0.002, I^2^ = 75.0%) (Figure 3).

### 3.5. Results of Synthesis: Efficacy Profile

**Figure 4:**
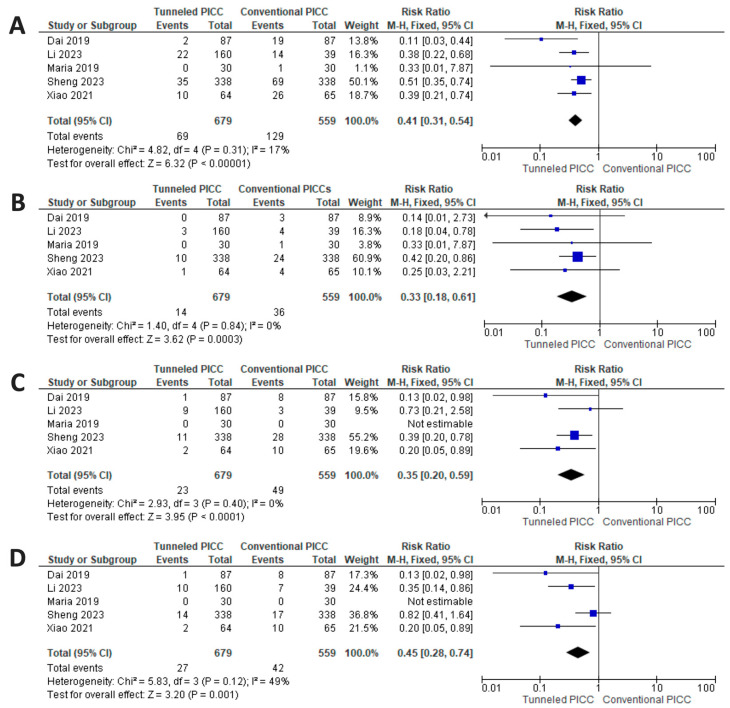
Results of synthesis. (**A**) significant reduction in the risk of catheter failure for tPICCs regarding all late complications (infection triggering catheter removal, CRT, and dislodgment). (**B**) Reduced risk for tPICCs for infection triggering catheter removal. (**C**) Reduced risk factor for tPICCs regarding CRT. (**D**) Reduced risk for tPICCs for risk of dislodgment [31,32,33,34,35].

Pooling all late complications (infection triggering catheter removal, CRT, and dislodgment), there is a significant reduction in the risk of catheter failure for tPICCs compared to cPICCs (*p* < 0.001; RR 0.41 [0.31–0.54] CI 95%), with a fixed model used due to non-significant between-study heterogeneity (*p* = 0.31, I^2^ = 17.0%) (Figure 4). The NNTB is 7.74 (5.9–11.27) CI 95%. 

The summary estimate of the infection risk shows a significant reduction in favor of tPICCs (*p* < 0.001; RR 0.33 [0.18–0.61] CI 95%), with a fixed model used due to non-significant between-study heterogeneity (*p* = 0.84, I^2^ = 0%) (Figure 4). The NNTB is 22.54 (15.22–45.7) CI 95%. 

A reduced risk factor for tPICCs (*p* < 0.001; RR 0.35 [0.20–0.59] CI 95%) can be noted regarding the risk of CRT, with a fixed model used due to non-significant between-study heterogeneity (*p* = 0.40, I2 = 0%) (Figure 4). The NNTB is 18.59 (12.53–36.03) CI 95%.

Regarding the risk of dislodgment, the analysis shows a reduced risk for tPICCs (*p* = 0.001; RR 0.45 [0.28–0.74] CI 95%), with a fixed model used due to significant between-study heterogeneity (*p* = 0.12, I^2^ = 49%) (Figure 4). The NNTB is 28.27 (16.4–102.4) CI 95%.

**Figure 5:**
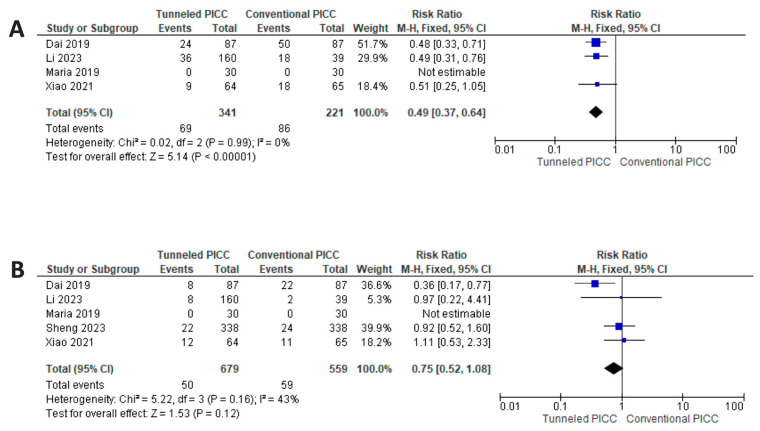
Results of synthesis. (**A**) Reduced risk for tPICCs for wound oozing. (**B**) Reduced risk for tPICCs for risk of MARSI [31,32,33,34,35].

When evaluating secondary outcomes, the analysis shows a reduced risk for tPICCS for wound oozing (Figure 5) (*p* < 0.001; RR 0.49 [0.37–0.64] CI 95%), with an NNTB of 5.35 (3.83–8.87) CI 95%, with a fixed model used due to non-significant between-study heterogeneity (*p* = 0.99, I^2^ = 0%,). Moreover, the analysis shows a trend in favor of tPICCs (*p* = 0.12; RR 0.75 [0.52–1.08] CI 95%) regarding MARSI (Figure 5), with a fixed model used due to non-significant between-study heterogeneity (*p* = 0.16, I^2^ = 43%).

## 4. Discussion

Although there is limited literature about the use of tunneling techniques for the placement of brachial access devices, recent data from the five RCTs reviewed suggest growing interest in the topic. To the best of the authors’ knowledge, this is the first review and meta-analysis of the tunneling of PICCs.

This meta-analysis shows the non-inferiority of tPICCs compared to cPICCs regarding safety profile. First, there are no significant differences when utilizing tPICCs compared to the traditional technique in terms of procedure success (*p* = 0.62) and the occurrence of nerve or artery injuries (*p* = 0.62). Furthermore, while there is a significant increase in procedural bleeding and procedure duration with the use of tPICCs compared to cPICCs (*p* = 0.02 and *p* < 0.001, respectively), it is noteworthy that the difference in bleeding volume between tPICCs and cPICCs is negligible (0.23 milliliters [0.04–0.42]), as is the growth of the procedure duration, which increases by 3.06 min (2.56–3.57).

Based on these results, tunneling procedures in brachial access do not cause significant bleeding or prolong procedural time. Moreover, the increased blood loss and time lost during the insertion are widely overcome by the advantages that the tunneling technique offers in the catheter dwell time. In practical terms, the safety values observed in this meta-analysis suggest that brachial access tunneling is a procedure with a medium risk of bleeding, consistent with the Gavecelt consensus statement [36].

According to the data, the risk of infection can be reduced by creating a subcutaneous tunnel (interestingly, the risk of infection does not increase when this step is added to the procedure). This is likely because the puncture site is separate from the exit site, and the subcutaneous route provides additional protection from contamination. Moreover, the exit site could be positioned through tunneling in a less contaminated bacterial area, as even recently reported in the Femoral Inserted Central Catheter with an exit site in the mid-thigh area [37,38]. 

tPICCs seem to be safer than cPICCs in terms of CRT, which has been identified as one of the most relevant complications of PICC implantation in previous studies [2,39]. Using the subcutaneous tunnel technique, the inserter can create a puncture site near or inside the armpit (where vessels are usually larger than in the arm), reducing the risk of venous thrombosis by optimizing the vein-to-catheter ratio. This is confirmed by the works of Xiao [32] and Dai [31], where a statistical difference between vein diameter at the venipuncture site and the exit site (*p* = 0.001 for both) is reported. In all the RCTs, PICCs with a French diameter of 4 were used. Nevertheless, tunneling techniques could provide favorable outcomes for multi-lumen catheter insertion in patients initially considered unsuitable due to the heightened risk of thrombosis [40,41]. However, to substantiate this hypothesis, it is imperative to carry out new well-designed RCTs.

The reduced risk of dislodgement in tPICCs compared to cPICCs could be explained by creating a subcutaneous tunnel, stabilizing the catheter, and offering increased resistance to possible tractions. However, as suggested by Sheng et al. [34], tunneling alone is insufficient to protect the catheter from the risk of dislodgment. However, it must be integrated with other tools, such as tissue glue and subcutaneous anchoring devices. 

Tunneling is often thought to be a risky maneuver with a higher chance of bleeding complications. However, post-insertion oozing in this meta-analysis is less common with tunneling than cPICCs (*p* < 0.001). This could be due to various factors, such as the hydro-dissection of subcutaneous tissues, different closures of the venipuncture site, and tunnel length. Although oozing from the exit site was observed, no bleeding from the venipuncture site was reported in any of the studies. Arm tunneling techniques could be considered less invasive, and additional safety measures like hydro dissection, using a blunt tunneler, and proper suturing can further reduce risks. However, to confirm this hypothesis, more well-designed RCTs are needed. It is important to note that all the insertions included in this meta-analysis were performed in oncological outpatients without bleeding disorders.

Regarding MARSI, tunneling shows a favorable trend (even if it does not reach significance). In particular, the data indicate that creating a subcutaneous tunnel (inserting an extra step inside the implant procedure) does not increase the MARSI risk.

Concerning the technique of creating a subcutaneous tunnel, Xiao [32], Li [33], and Sheng [34] use a metallic tunneler, while Maria [35] and Dai [31] use a peripheral cannula. There were no differences in the outcomes, such as recommending one method over the other. To obtain a more comprehensive and precise analysis, conducting more RCTs that focus on assessing the efficiency of different equipment would be beneficial. The information and data derived from these trials could assist in guiding and informing future decision making regarding this matter. When considering materials, both silicon and power-injectable polyurethane devices were used; no complications related to materials (ruptures, embolization) were observed.

The Recommended length of the tunnel still needs to be discovered in the literature, but based on Li et al.’s RCT about this issue, a length over 4 cm should be performed to obtain a reduction in complication risk [33].

Various suturing techniques were used to suture the skin at the venipuncture site: traditional suturing, tissue adhesive, and reinforced adhesive skin closures. Although more specific research should be conducted, all techniques have proven effective in reducing oozing and sealing the site.

### Limitations

The meta-analysis we conducted presents some limitations. The paucity of studies in the literature could be one of them, since just five publications and a total of 1238 patients were recruited through our study research method. Also, both studies by Xiao [32] and Dai [31] would have needed a larger sample size to evaluate less frequent complications such as CRT or catheter-related infection and unlikely wound oozing, which is a relatively frequent complication. Furthermore, both studies by Xiao [32] and Dai [31] were carried out by the same authors and in the same institution over two timeframes so that the same operators presumably inserted the PICCs. Although a distinct tunneling technique was used in the two studies, local factors and practices may have impacted our meta-analysis findings. 

Maria et al. [35] suffer limitations such as the lack of definition of a priori endpoints or how the follow-up was conducted.

The excellent homogeneity of the population observed (all studies were carried out on oncological patients without bleeding disorders) does not allow us to express opinions regarding the use of tunneling techniques in other populations. An unsolved question is that pertaining to the effect of tunneling in non-cancer inpatients where the dwell time of the catheter is usually shorter, and the likely tunnel protection is still unknown.

## 5. Conclusions

The results of our meta-analysis suggest that tPICCs are safe concerning intra-procedural bleeding, overall success, and procedural time and are also effective in terms of early and late complications. TPICCs significantly reduce the risk of infections, catheter-related thrombosis, and dislodgment in cancer patients. The benefits of tunneling in the inpatient population are still being determined. Further studies with larger populations are warranted to address these issues. 

## Figures and Tables

**Table 1:** Study characteristics.

Author, Years	Country, Type, and Time of Enrollment	Tunneling Tech.	Skin Closure	PICC Dressing *	Definition of Wound Oozing	Definition of Thrombosis	Definition of Infection	Definition of Dislodgement
Dai et al., 2019 [31]	China Monocentric from July 2018 through May 2019 (11 months)	Large peripheral cannula 14 G	Wound closure strip	Gauze and transparent membrane	Wound oozing as an important outcome was recorded when oozing lasted more than 24 h. Grade 1 was a small amount of oozing lasting for 2–3 days, Grade 2 was oozing lasting for 4–5 days, and Grade 3 was oozing lasting more than 6 days	Venous thrombosis was classified as symptomatic or asymptomatic and confirmed as having an association with the PICC or occurring within 5 days of extubation	Infections resulting from the use of catheters were defined according to national infection-control guidelines.	Catheter dislodgement was recorded when the tip moved more than 2 cm.
Xiao et al., 2021 [32]	China Monocentric from July 2019 through January 2020 (7 months)	Blunt tunneler	Wound closure strip	Gauze and transparent membrane	Oozing that lasted >24 h after placement. Classified into three grades according to severity: Grade 1 (bleeding lasting for 2 to 3 days), Grade 2 (bleeding lasting for 4 to 5 days), and Grade 3 (bleeding lasting >6 days).	The presence of an intraluminal thrombus as confirmed by color Doppler ultrasound. Classified as symptomatic or asymptomatic (symptomatic thrombosis was diagnosed when symptoms occurred).	Defined according to the Centers for Disease Control and Prevention and classified as local infection or central line-associated bloodstream infection.	Exposed portion of PICC prolapsed by >2 cm.
Li et al., 2023 [33]	China Monocentric from March 2021 through August 2021 (6 months)	Blunt tunneler	Nylon sutures	Gauze and transparent membrane	Oozing that lasted for more than 24 h.	Venous thrombosis was identified by pain and swelling of the arm and confirmed by B-mode ultrasound	Infections included local skin infections and CLABSI, which were diagnosed by clinical physicians and confirmed by blood culture results.	The catheter shifted more than 2 cm
Sheng et al., 2023 [34]	China Multicenter (three hospital) from August 2011 through December 2021 (5 months)	Blunt tunneler	Octyl cyanoacrylate skin adhesive	Glue and transparent membrane	N/A	CRT was confirmed by ultrasound or CT examination showing the presence of a thrombus in the vein with a catheter	Infections were defined according to Infectious Diseases Society of America criteria	Catheter malposition was defined as exposed length prolapsed ≥5 cm
Maria et al., 2019 [35]	Greece Monocentric from August 2014 through February 2015 (7 months)	Large peripheral cannula 14 G	Nylon sutures	Glue and transparent membrane	N/A	N/A	N/A	N/A

Legend: PICC: Peripherally Inserted Central Catheter; *: dressing in first 48 h.

## Data Availability

The study data will be made available upon request to the corresponding author.

## Supplementary Materials

The following supporting information can be downloaded at: https://www.mdpi.com/article/10.3390/nursrep14010035/s1, Table S1 research strategy.

## Public Involvement Statement

There was no public involvement in any aspect of this research.

## Guidelines and Standards Statement

Our systematic review and meta-analysis was conducted following the Preferred Reporting Items for Systematic Reviews and Meta-analysis (PRISMA) guidelines [15].
