# Supplementary material for: Brachial Tunneled Peripherally Inserted Central Catheters and the Risk of Catheter Complications: A Systematic Review and Meta-Analysis
Source: Nurs Rep. 2024 Feb 18;14(1):455–67. doi: 10.3390/nursrep14010035 (PMC10885060; doi:10.3390/nursrep14010035)
Supplement: Supplementary file 1 [file nursrep-14-00035-s001.zip › nursrep-2657475-supplementary.pdf]

NO	search	Results retrived
1	Peripherally Inserted Central Catheter	3750
2	PICC	2013
3	#1 OR #2	4052
4	tunnel	71459
5	subcutaneous tunneling	1748
6	#4 OR #5	71857
7	#3 AND #6	564
8	#7 AND randomized controlled trial	236
